# Embryo location in the uterus during embryo transfer: An *in vitro* simulation

**DOI:** 10.1371/journal.pone.0240142

**Published:** 2020-10-05

**Authors:** Jinqiu Mo, Qing Yang, Lan Xia, Zhihong Niu

**Affiliations:** 1 School of Mechanical Engineering, Shanghai Jiao Tong University, Shanghai, China; 2 Reproductive Medical Center, Obstetrics and Gynecology Department, Ruijin Hospital Affiliated with the Medical School of Shanghai Jiao Tong University, Shanghai, China; Infertility Unit, ASST Lariana, ITALY

## Abstract

**Objective:**

To evaluate the location of transferred embryos under various parameters during embryo transfer in *in vitro* fertilization (IVF) by applying an *in vitro* experimental model for embryo transfer (ET).

**Methods:**

Mock ET simulations were conducted with a laboratory model of the uterine cavity. The transfer catheter was loaded with a sequence of air and liquid volumes, including development-arrested embryos donated by patients. The transfer procedure was recorded using a digital video camera. An orthogonal design, including three independent variables (uterine orientation, distance of the catheter tip to the fundus, and injection speed) and one dependent variable (final embryo position), was applied.

**Results:**

The uterine cavity was divided into six regions. The distribution of the transferred matter within the uterine cavity varied according to the uterine orientation. Medium speed-injected embryos were mostly found in the static region while fast- and slow-speed injected embryos were mostly found in the fundal region and the cervical-left region, respectively. The possibility of embryo separation from the air bubble increased from 11.1% in slow injection cases to 29.6% and 48.1% in medium and fast injection cases, respectively.

**Conclusion:**

The experimental model provides a new method for investigating ET procedures. Fast injection of embryos into a retroverted uterus may be more likely to result in embryo separation from the air bubble.

## Introduction

Many aspects of the *in vitro* fertilization (IVF) procedure have progressed significantly over the past 30 years. However, the technique of embryo transfer (ET), a simple yet critical element of IVF, has remained relatively unchanged. The goal of a successful ET is to deliver embryos to a location in the uterus where the probability of implantation is maximized. After cleansing the cervix, a transfer catheter loaded with embryos is inserted through the cervical canal and advanced into the uterine cavity where the embryos are deposited. The transfer catheter is usually loaded using a ‘three-drop technique’, in which the drop of medium containing the embryo(s) is separated from the preceding and following drops of medium with an air bubble [1]. After the embryo(s) have been slowly released into the uterine cavity, the catheter is withdrawn and handed to the embryologist, who inspects it for retained embryo(s).

Technical parameters, such as catheter placement, injection speed, catheter withdrawal, location of the catheter tip, and location of the air bubble may influence the success of the IVF procedure [2–4]. However, the effects of these factors on the outcome of pregnancy are controversial [5, 6], as the movement and location of embryos in the uterine cavity are not visible.

The difficulty associated with studying ET techniques is that embryos cannot be labeled, making it impossible to track their movement and deposition in the uterine cavity. Ultrasound guidance is however, particularly helpful for visualizing the transfer catheter, air bubbles, and endometrial cavity [7]. Generally, the air bubbles are believed to indicate the final position of the embryos and can, therefore, be used as a marker for embryo position [8, 9], but there is no tangible evidence to support this theory.

*In vivo* investigation of the mechanical aspects of the ET procedure is limited due to medical and ethical considerations. Eytan et al. [10] reported laboratory and computational models that enabled the investigation of the ET procedure *in vitro*. Within the laboratory model, dispersion of the transferred medium, which was considered a predictor of the location of embryos, could be recorded and analyzed. Considering that the trajectory of the medium may be inconsistent with that of the embryo, we improved the model proposed by Eytan et al. by loading human embryos in a transfer catheter and observing embryo deposition sites rather than liquid dispersion alone. The aim of our study was to investigate post-ET positioning of embryos within a mock uterine cavity to ascertain the various parameters of ET and their relationship with the dispersion of transferred matter. Additionally, we sought to obtain direct evidence for the reliability of using the location of the transferred air bubble to identify the position of the embryos.

## Materials and methods

### Experimental apparatus

**Fig 1:**
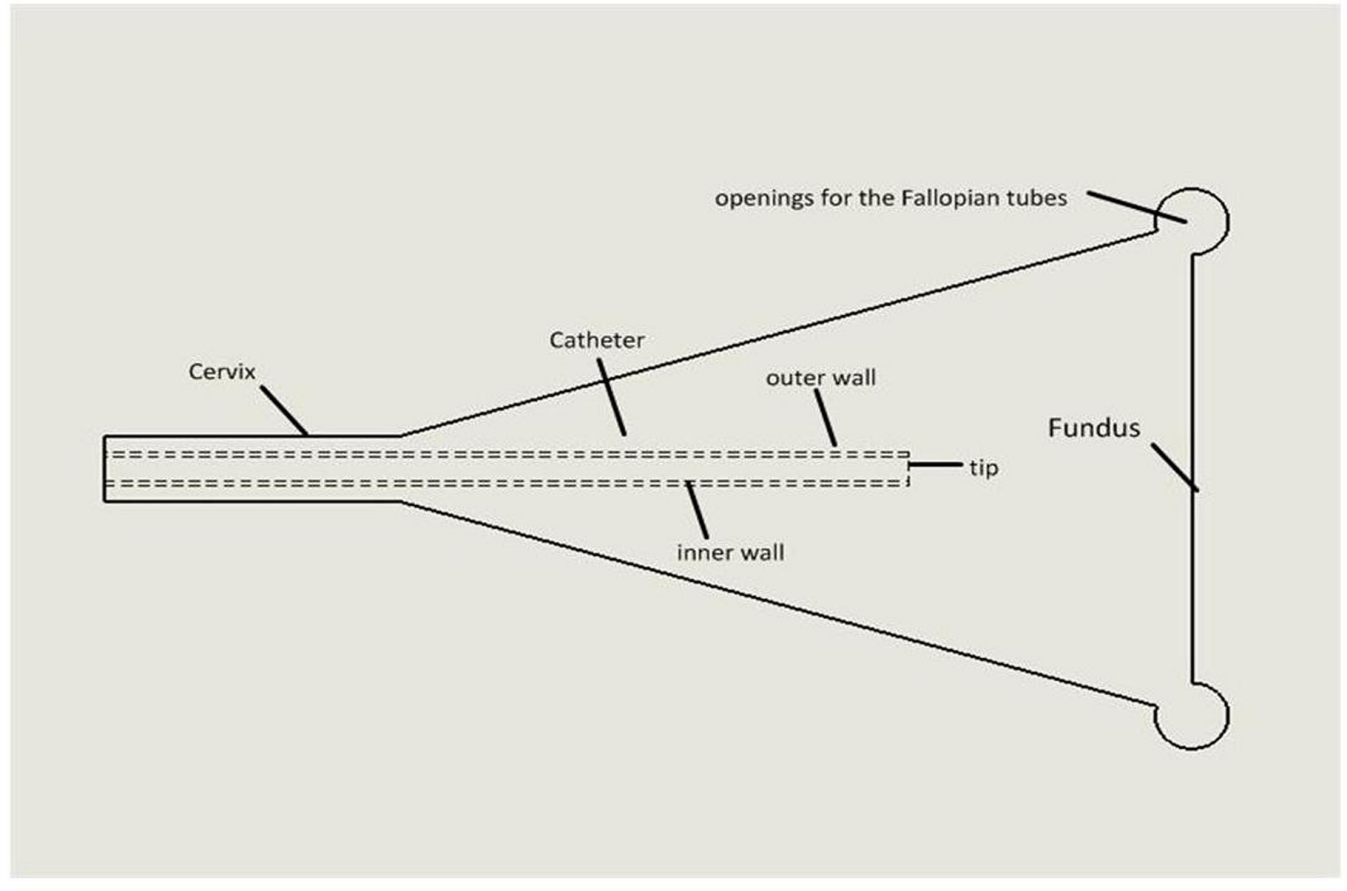
Schematic illustration of the in vitro embryo transfer model.

**Fig 2:**
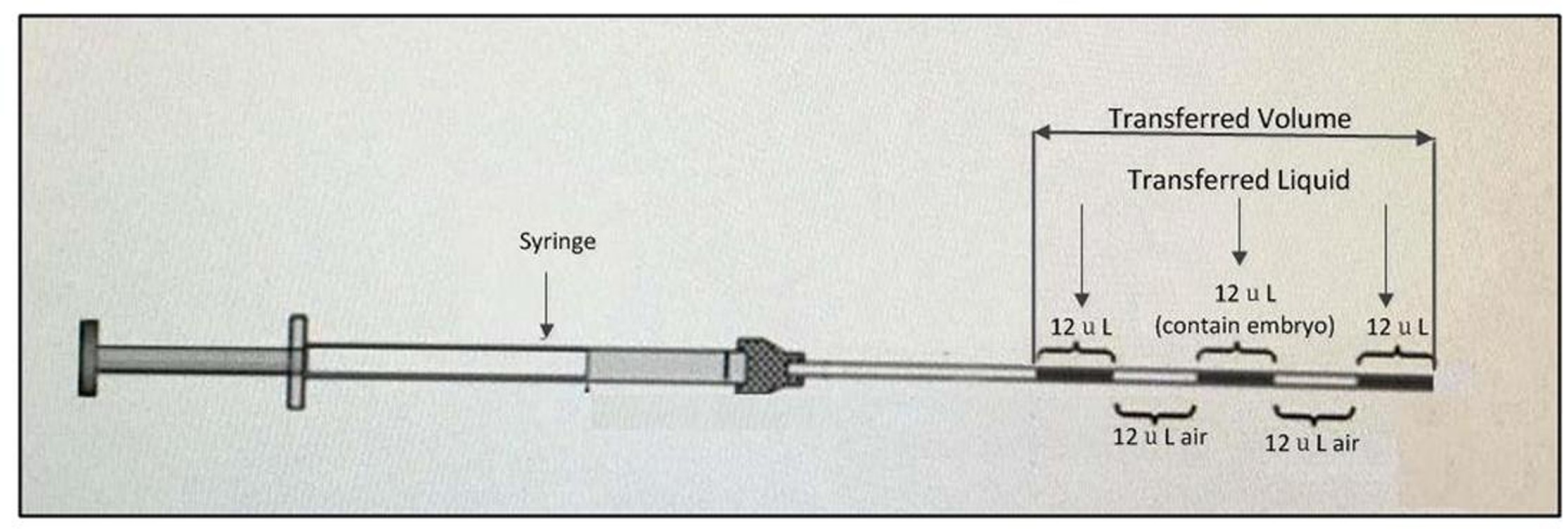
The catheter load with a sequence of air and liquid volumes.

The *in vitro* experimental system for simulating *in utero* ET has been detailed by Eytan et al [10, 11]. We set up a mock system based on their published studies. Briefly, the system consisted of a rigid and transparent uterine model that reproduces the inner cavity of the uterus, a loaded catheter that is driven by a computerized micro-injection pump, and a plate with a digital video camera. The uterine cavity model comprises two transparent polyvinyl chloride boards separated by a 2.5 mm thick rubber pad with two 0.3 mm openings to simulate the fallopian tubes. This model is filled with glycerine to mimic the highly viscous uterine fluid, and the internal pressure can be measured via a sensor connected through the pseudo-fallopian tube openings (Fig 1). Our uterine model was installed on a plate, which could be inclined between 0° and 60° above or below the horizontal plane to simulate the various uterine orientations of patients during a real ET procedure. The Frydman catheter (Laboratoire CCD, Paris, France) was inserted into the uterine model through a rubber sealing at the cervical opening for transcervical transfer of embryos. The catheter was loaded with a sequence of air and liquid volumes using the following order from the catheter tip (Fig 2): 12 μL liquid, 12 μL air, 12 μL liquid (which contained embryos as in a real ET), 12 μL air, and 12 μL liquid. The transferred liquid used was P-1 Medium (Irvine Scientific®, CA), with a viscosity of 2 cP. A peristaltic pump system was selected because of its excellent ability to pump small volumes of matter and generate a standardized injection speed. The speed of injection was controlled by a computer and the transferred liquid was colored with 10 mg of bromophenol blue dissolved in 10 mL of 10 mM Tris, pH 8. The density and dynamic viscosity of glycerine in the uterine model were ρ = 1,236.25 kg/m^3^ and μ = 0.799 kg/(m‧s), respectively. This density is similar to that of uterine fluid [12]. A digital video camera (SONY, HDRCX900E) and adequate lighting were installed in the system to acquire and record the spreading patterns of transferred liquids at a rate of 100 fps.

The study was approved by the Ethics Committee of Ruijin Hospital, and written informed consent was obtained from all subjects. All experiments were performed in accordance with the Declaration of Helsinki. Authors had access to information that could identify individual participants during or after data collection.

## Experimental protocol

### Experimental design

We aimed to study three main parameters involved in ET: uterine orientation, injection speed, and distance from the catheter tip to the fundus. Therefore, we applied an orthogonal design, including three independent variables: uterine orientation (A), distance from the catheter tip to the fundus (B), and injection speed (C), and one dependent variable: the final position of the embryo in the uterine cavity. The levels and factors of the orthogonal design are listed in Table 1. In our study, factor A (uterine orientation), had nine levels while the two other factors had three levels each. Normally, in a set up of three factors with nine, three, and three levels, respectively, 81 (9×32) experiments should be conducted. Unlike a factorial design where all possible combinations are tested, the orthogonal table employs a minimal number of trials by testing pairs of combinations, which saves both time and resources.

**Table 1:** Experimental design based on the L27 (3×9) orthogonal array and the experimental results.

**TEST NUMBER**	**FACTORS LEVEL**		
	A	B	C
	Utero angel	Depth(mm)	Speed(ul/s)
**1**	30°	5	2
**2**	30°	10	6
**3**	30°	15	3
**4**	45°	5	3
**5**	45°	10	2
**6**	45°	15	6
**7**	60°	5	6
**8**	60°	10	3
**9**	60°	15	2
**10**	-30°	5	6
**11**	-30°	10	3
**12**	-30°	15	2
**13**	-45°	5	3
**14**	-45°	10	2
**15**	-45°	15	6
**16**	-60°	5	2
**17**	-60°	10	6
**18**	-60°	15	3
**19**	-15°	5	2
**20**	-15°	10	6
**21**	-15°	15	3
**22**	0°	5	3
**23**	0°	10	2
**24**	0°	15	6
**25**	15°	5	6
**26**	15°	10	3
**27**	15°	15	2

^a^ 15°,30°,45 and 60° indicates an anterior uterus; -60°,-45°,-30,°-15°indicates a posterior uterus; 0°indicates a horizontal uterus.

The levels and factors of the orthogonal design, according to the L27 orthogonal array, are listed in Table 1.

### ET procedure simulation

The mock ET simulation was based on a protocol, which is similar to the real ET procedure, as performed in our IVF unit. The catheter was loaded with a series of air and transfer liquids (P-1 Medium), as described above. Because the aim of this research was to study post-ET positioning of transferred embryos, we loaded the transfer catheter with development-arrested embryos donated by patients in June 2019, when we performed the simulation study. As seen in Table 1, 27 cases of different uterine orientations, catheter-fundus distances, and injection speeds were simulated, with each case simulated three times to ensure reproducibility of the results. Subsequently, the catheter was gently removed from the uterine model through the “cervix,” 30 seconds after the end of the injection. Thereafter, the position of the embryos was checked under a stereomicroscope (NIKON, SMZ800N, Minato, Tokyo, Japan). The entire procedure, from the beginning of the injection to the withdrawal of the catheter, was recorded using a video camcorder.

### Divisions of the uterine cavity

**Fig 3:**
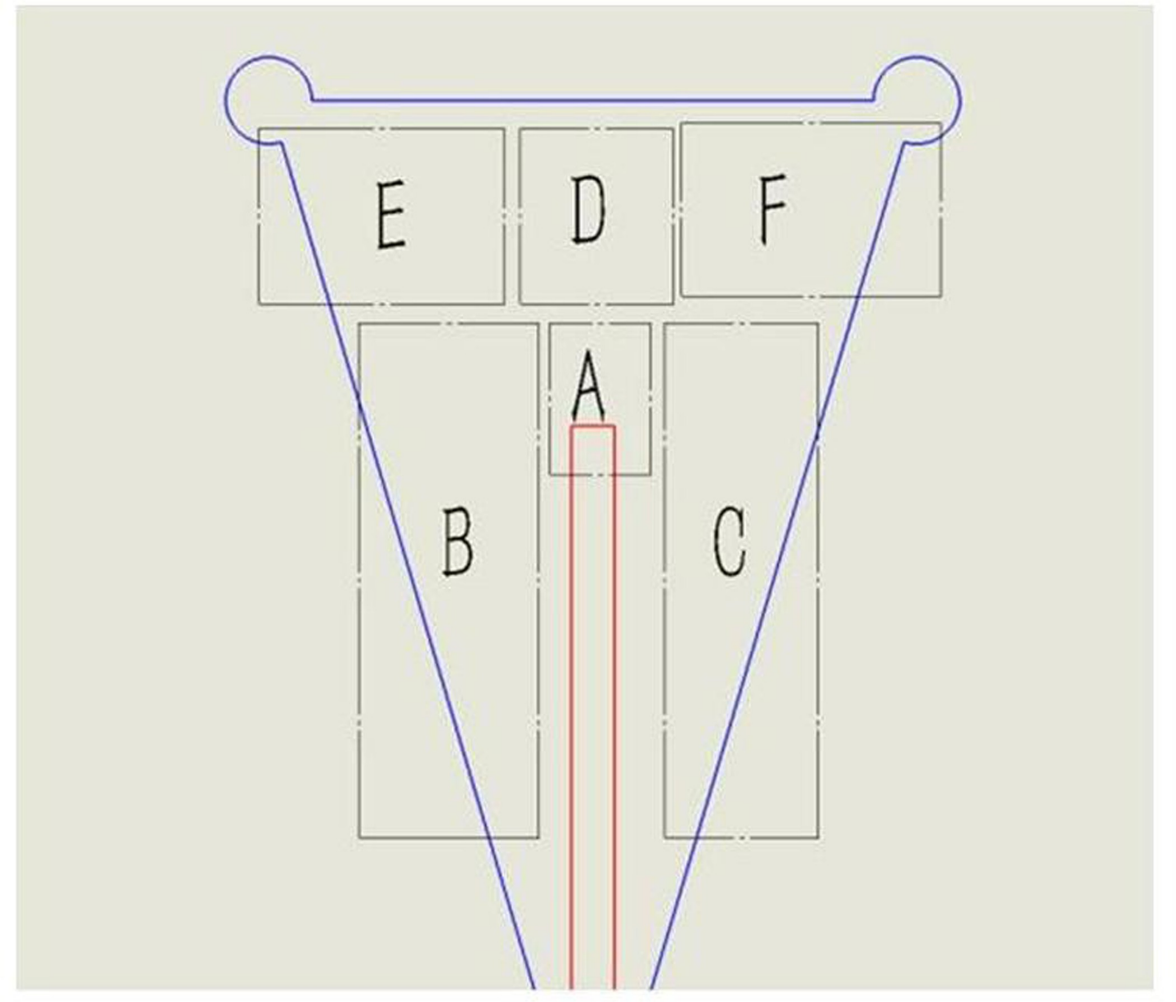
Schematic illustration of the division of embryo locations in the uterine cavity.

To describe the location of the embryo in the uterine model, we divided the cavity into six regions (Fig 3 and Table 2). Generally, the endometrium near the fundus (static and fundal areas) is considered a more suitable location for embryo implantation. Embryo flash movement toward the cervix after ET (cervical-left and cervical-right areas) may cause a decrease in the pregnancy rate and an increased risk of expulsion from the uterine cavity [13, 14]. The area in close proximity to the fallopian tube openings (horn-left and horn-right areas) is also an inappropriate location for embryo implantation because of the risk of an ectopic pregnancy.

**Table 2:** Embryo location area division.

**Area**	**Description**	**Observation under Ultrasound**
**A**	Within 3 mm around the catheter tip	embryo flash remaining almost static from its original position
**B**	Left side of the catheter, close to the cervix	embryo flash migration towards the cervix from its original position
**C**	Right sides of the catheter, close to the cervix	
**D**	Between area A and the fundus	embryo flash migration towards the fundus from its original position
**E**	Between area D and the simulated left “fallopian tube” opening	embryo flash migration towards the two fallopian tubes from its original position
**F**	Between area D and the simulated right “fallopian tube” opening	

## Results

The injection speed was defined as fast (6 μL/s), medium (3 μL/s), and slow (2 μL/s), and the catheter-fundus distance as close (5 mm), medium (10 mm), and far (15 mm).

### ET mock procedure

#### Expansion pattern of transferred matter

**Fig 4:**
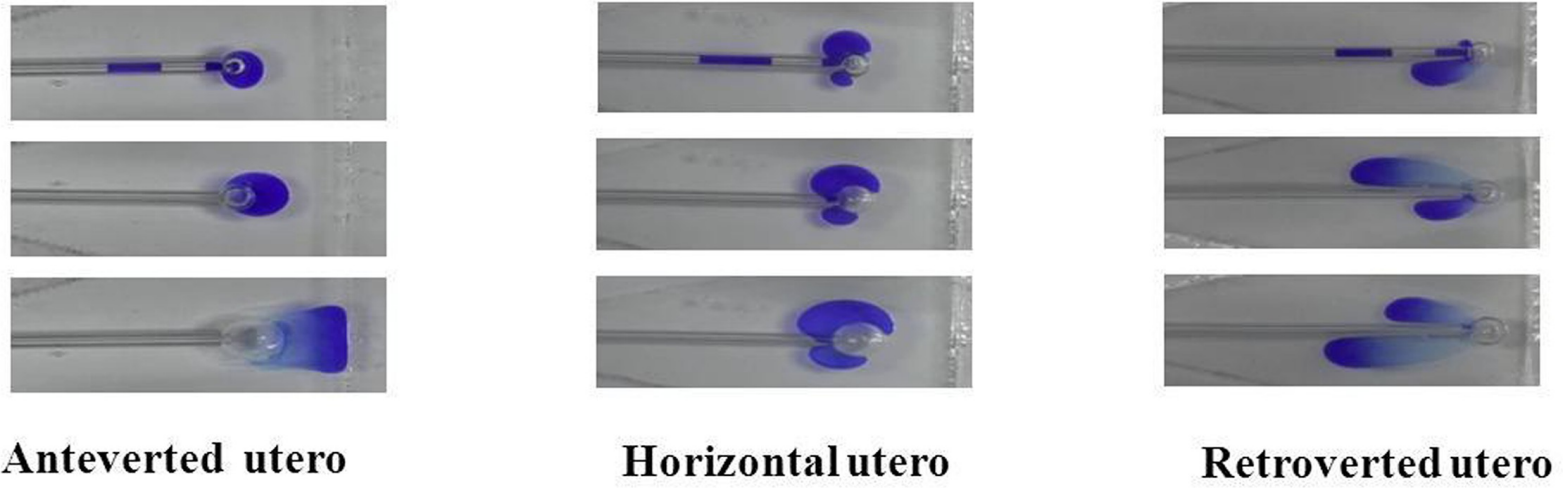
Transferred matter expansion shapes in the anteverted, horizontal, and retroverted uterus of the laboratory ET model.

As air came off the catheter tip (immediately after the onset of injection), it formed two small volumes of air that merged to become one large air bubble, which was attached to the catheter tip as the injection proceeded. The transferred liquid (indicated by the blue marks in Fig 4) spread away from the catheter tip, expanded, and formed various shapes in the different uterine orientation models. In the anteverted uterus, the diffused liquid moved toward the fundus as a whole, forming a trapezoid in the area between the catheter tip and the fundus. In the horizontal and retroverted uterus, the diffused liquid moved toward the cervix immediately after spreading from the catheter tip, forming two separate fluid areas, and expanding on both sides of the catheter tip. Although the transferred liquid mainly spread around the catheter tip in the horizontal uterus, it diffused backwards toward the cervix in the retroverted uterus.

#### The position of embryos in different cases

**Fig 5:**
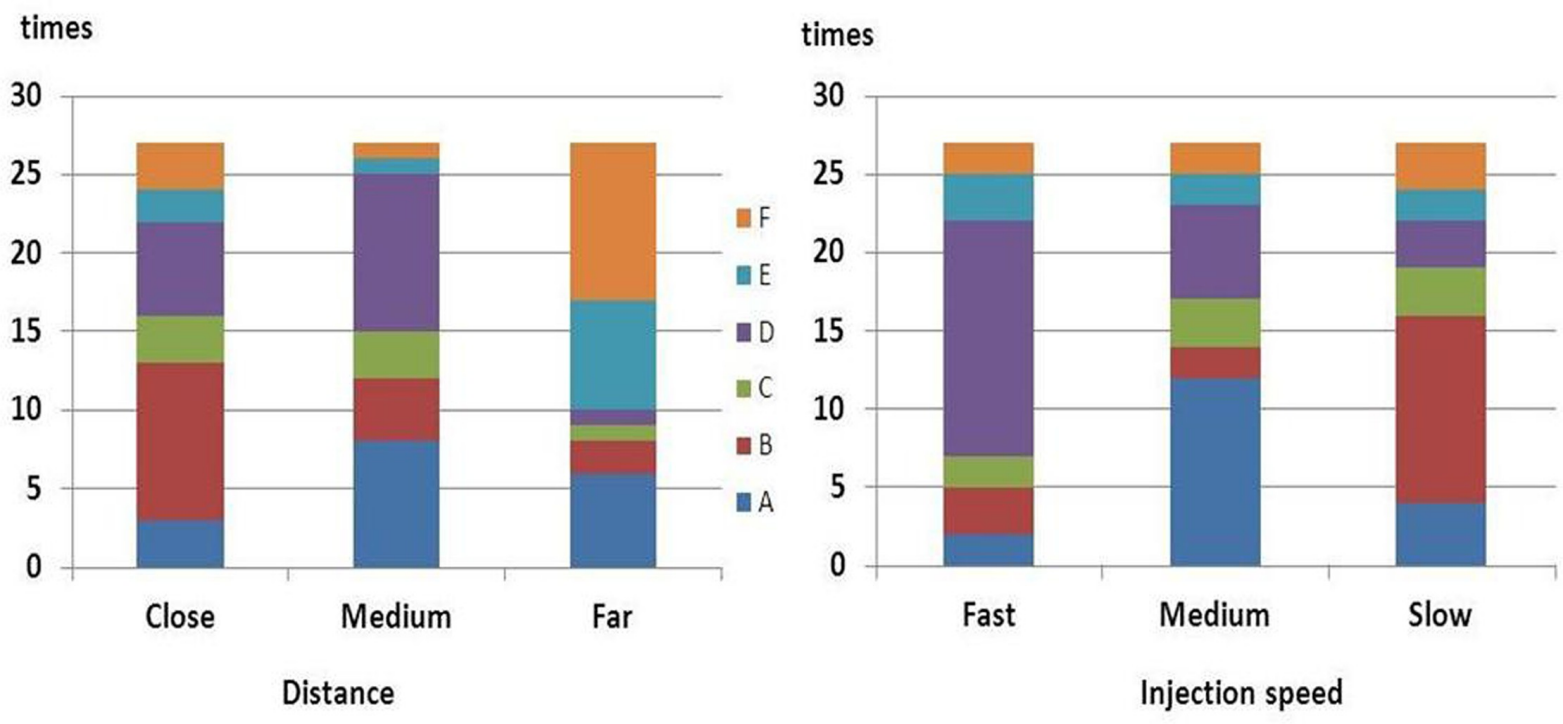
Embryo locations under different injection speeds and catheter tip-fundus distances in the laboratory embryo transfer model.

We performed the mock procedure three times in each case, the results of which are presented in Fig 5. To make the results clearer, we set the catheter-fundus distance at 10 mm when observing the effect of injection speeds, and the speed at 3 μL/s when investigating the effects of catheter-fundus distances.

Our results indicated that medium speed-injected embryos were mostly found in the static area (12/27), followed by the fundal area (6/27). Fast speed-injected embryos had increased chances of being deposited in the fundal area (15/27). Slow-speed injected embryos were mostly found in the cervical-left area (11/27). Under medium injection speed, a medium catheter-fundus distance resulted in the highest frequency of embryo deposition in the fundal area (10/27), followed by the static area (8/27). However, in cases of close and far distances, embryos were mostly located in the cervical-left area (10/27) and the horn-right area (10/27), respectively.

#### Separation of the embryo from the air bubble

**Fig 6:**
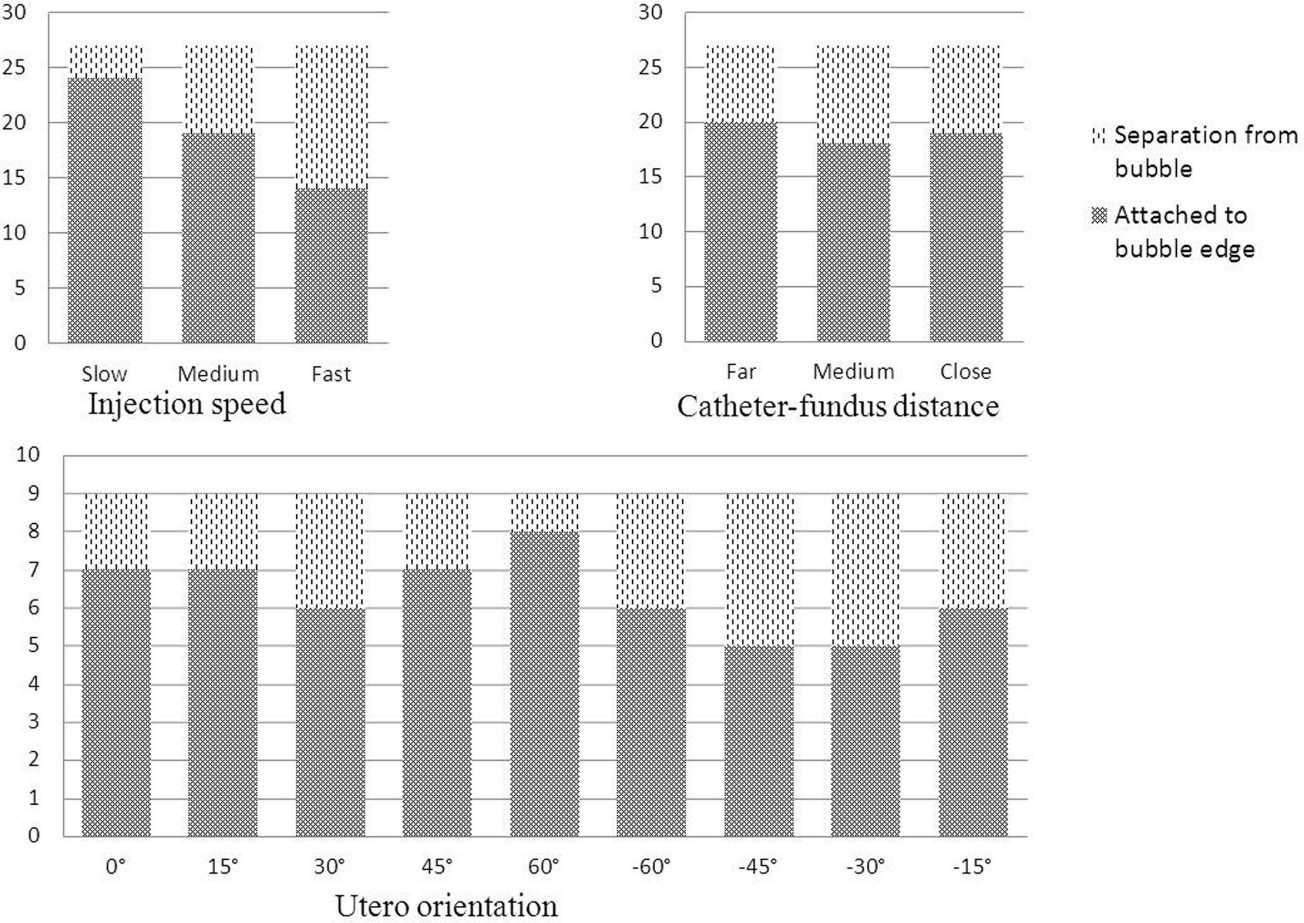
The separation of embryos from the air bubble under different injection speeds and catheter tip-fundus distances in the laboratory ET model.

Fig 6 reflects the proportion of embryos that separated from the air bubbles. Overall, the inconsistency between the location of the air bubble and embryo was 29.6%. The possibility of embryo separation from the air bubble increased from 11.1% in slow injection cases to 29.6% and 48.1% in medium and fast injection cases, respectively. However, the possibility of the embryo attaching to the edge of the air bubble was almost the same among the three catheter-fundus distances, varying between 70.4% and 77.8%. In the anteverted uterus, the possibility of the embryo deviating from the edge of the air bubble ranged from 11.1% to 33.3% according to the injection speed and catheter tip-fundus distance. In the retroverted uterus, however, it was raised to 33.3% to 44.4%.

## Discussion

The ultimate goal of ET is to place the embryo in an area of the uterine cavity where the probability of implantation is highest. It is generally accepted that the fundal endometrium is suitable for implantation because of the expression of important factors related to implantation, and there is a tendency for lower endometrial wavelike activity and higher endometrial tissue blood flow in the fundal endometrium [15, 16]. However, a high fundal ET with a catheter tip-fundus distance that is too close to the uterine horns has an increased risk of resulting in an ectopic pregnancy as the embryo moves toward the uterine horns. It has been speculated that catheter withdrawal could result in movement of the air bubble toward the cervix, which could expel embryos from the uterus [4, 17, 18]. Thus, a major objective of a successful ET protocol should be to transfer embryos far away from the catheter tip, in a manner that ensures that they will be pushed to an appropriate distance from the fundal wall and will remain there. In the present study, we defined the fundal area as the best area for embryo deposition and the static area as the second-best area. A final landing point in the horn-left or horn-right areas was unfavorable, as it is susceptible to an ectopic pregnancy. The cervical-left or cervical-right regions were also unfavorable deposition sites with the risk of embryos leaving the uterus, as these sites were too close to the cervix.

### Transferred matter

Although the protocol of catheter loading varies between different IVF units, there are similarities in the use of small transferred volumes, which comprise a sequence of air and liquid contents, as this would ensure that embryos do not stick to the catheter wall during injection [19].

Yaniv et al. [20] reported that the intrauterine fluid field and embryo transport patterns were strongly affected by the closed fundal end, through 2-dimensional computational modeling of the uterus. The results of our study revealed that the spreading pattern of the transferred matter within the uterine cavity was dependent on the uterine orientation under a fixed injection speed and catheter-fundus distance. A 24-μL volume of air in the transfer catheter was responsible for the formation of one air bubble. During the injection procedure, the air bubble developed and remained close to the catheter tip due to the surface tension of the viscous liquid, while the two liquid volumes merged from the onset of the injection and were transported in different directions. In a horizontal uterus, the transferred liquid remained mostly stationary around the catheter tip, while the transferred fluid moved toward the fundus and cervix in the anteverted and retroverted uteruses, respectively. Based on these results, the mock ET model suggested that the orientation of the uterus may greatly affect the dispersion style of the transferred matter. The forces of buoyancy became dominant as the inclination of the uterus increased, dragging the transferred matter toward the fundus (anteverted uterus) or cervix (retroverted uterus). During the ET procedure, the angle of inclination of the uterine cavity determined the dynamic interactions between compression stresses from the surrounding intrauterine liquid, gravity forces due to its weight, and buoyancy forces from the intrauterine liquid. Thus, a major outcome of this study, and of previous studies [11, 21], is that dynamic transport of the transferred matter is dependent on the position of the sagittal cross-section of the uterine cavity with respect to the horizontal. Fıçıcıoğlu et al. [22] reported that the clinical pregnancy rate was remarkably reduced when the embryo migrated from its original position toward the cervix 60 minutes after ET. Thus, a patient-specific position is recommended during the ET procedure to ensure that the fundus is at the highest point above the horizontal.

### Distance

Usually, in an ET procedure, the catheter is positioned 5 ± 15 mm from the fundus, a position that ensures that the embryos reach the fundus for implantation [2, 4, 23]. Several studies have analyzed the influence of catheter position on pregnancy rate but their conclusions are controversial. Friedman et al. [1] reported an improved pregnancy rate when the ET was close to the fundus (<10 mm) using blastocyst-stage embryos. Tires et al. [24] reported that the pregnancy rate was highest when embryos were transferred between 10 mm and 20 mm from the fundus. Furthermore, Kovacs et al. [25] found that transfer depth did not affect implantation and pregnancy rates when ET was performed in the middle or upper-third of the uterus. Eytan et al. [11] explored the dispersion of the transferred volume in an *in vitro* laboratory model to ascertain the appropriate positioning of the catheter tip, either close to (<1 cm) or far (>2.5 cm) from the fundus. In close distance cases, the fundal wall interfered with the dispersion of the transferred volume and pushed it to the sides. Thrusting the catheter tip further toward the fundus (a distance of 5 mm) and injecting the medium at a high speed drove the transferred liquid out of the uterine cavity through openings representative of the fallopian tubes, an event that could lead to an ectopic pregnancy in the real-life setting. Contrary to the findings of Eytan et al. [21], we did not observe diversity in the dispersion of transferred matter between the close, medium, and far distance cases, but the location of the embryos showed discrepancies. Embryos transferred too close to the fundus were pushed toward the cervix, and those transferred at far distances had the highest frequency of deposition near the fallopian tube openings. The disparities between our results and those of Eytan et al. may be related to differences in injection speed and the loading pattern of embryos in the transfer catheter.

### Injection speed

The speed of the catheter load delivery is another uncontrolled parameter of ET since the procedure is performed manually and varies between different operators. Usually, most practitioners inject the transfer load slowly, based on the belief that this would avoid embryo transport to the fallopian tube, despite the lack of evidence to support this notion [26]. To our knowledge, no consensus exists regarding the speed of injection during ET. Eytan et al. [10] reported that the length of delivery varied between 1 s (e.g., injection in a single push) and 15 s (e.g., very gentle injection) in the same IVF unit. This variation in injection speed may also explain the variation in the position of air bubbles in the uterine cavity even after standardizing the catheter position in ultrasound-guided ET [27].

It has been suggested that fast injection leads to embryo transport to farther distances downstream the catheter tip, preventing embryo washout during catheter withdrawal [11]. When the injection speed was fast, rapid release of air created local instantaneous high pressure around the bubble. As the radius of the bubble increased, the forces of buoyancy also rapidly increased, while the surface tension decreased. This detached the bubble and pushed it toward the fundus more quickly than was observed during slow injection. Consistent with the above findings, the results of the present study indicated that in slow-speed injection, the chances of depositing an embryo in the area close to the cervix increased, increasing the risk of ejection from the uterus. Medium and fast injections were associated with a higher chance of embryo deposition in areas near the fundus, which are favorable sites for embryo implantation. However, fast injection also increased the shear stress, dynamic pressure, and difference in velocity of an embryo during ET [28]. The increased fluid shear stress imposed on the embryo can induce morphological changes and trigger apoptotic processes. It has been proven in a laboratory model that morphological changes in response to ET were most prevalent in mouse blastocysts exposed to a fast ET [29]. The mean apoptotic index was 52% in the fast ET group and 25% in the slow ET group. However, the definition of an appropriate injection speed varied according to the embryo loading style and the inner diameter and material of the ET catheter. In this study, the medium speed was set to complete 60 μL of transfer matter in 20 s (3 μL/s). Eytan et al. [11] defined medium speed as completing the transfer of a 110 μL load in 10 s (11 μL/s), but they used a different loading style.

### Air bubble

Sonographic guidance can be very instructive during the ET procedure because the embryo-containing medium is often bracketed by air, and the air-fluid interface is easily visualized via ultrasound. The embryos are sandwiched between air bubbles in the transfer catheter. This is usually referred to as the “double bubble sign” [30] or “transfer flash” [10]. Due to medical and ethical considerations, it is assumed in all studies involving ET techniques that the embryo flash can reflect the actual embryo position. Embryo flashes migrating toward the cervix are believed to denote an increased risk of embryo expulsion from the uterine cavity. To date, the majority of studies on the air bubble seem to suggest that pregnancy rates are highest when the air bubbles are closer to the fundus [19, 26]. Fıçıcıoğlu et al. [22] also reported that air bubbles moving toward the fundus 60 minutes after catheter withdrawal were associated with higher clinical pregnancy rates [22]. Notably, the association of air bubble location and embryo deposition seems controversial. Lambers et al. [26] noted that in ETs that resulted in pregnancy, the final position of the air bubbles was closer to the fundus than it was in ETs that did not result in pregnancy. However, in the same study, it was also observed that despite standardization of the transfer protocol, the final position of the transferred air bubbles was unpredictable. Saravelos et al. [31] reported that only 40.8% and 50.7% of embryos were implanted at the location where the air bubbles were visualized at 1 min and 60 min after ET, respectively. In the present study, the difference in the location of the air bubble and embryo was observed directly. We identified separation of the embryo from the air bubble in about 30% of cases, and there was increased disparity between their locations in the retroverted uterus than in the anteverted uterus. Additionally, fast injection resulted in more displacement of the embryos from the air bubble than slower injections. The density difference between the embryo and air bubble, the change in the surface tension of the air bubble, and the cavity pressure after catheter withdrawal may have resulted in the inconsistent moving track of the embryo and air bubble.

Based on our data, along with previous reports, we were inclined to suggest a re-evaluation of the clinical significance of using the location of air bubbles to indicate the position of embryos under ultrasound ET guidance. As the conformity between the two locations was dependent on many conditions, including uterine orientation and injection speed, further studies are required to better understand the significance of air bubbles in ET.

### Limitations

Our non-invasive method of simulating ET using a laboratory model provides a preliminary model for research on ET technology, but it is still relatively complex and is not commonly available. The uterine model in this study was rigid; therefore, it could not simulate uterine contractions, which are suggested to be the mechanism responsible for pushing embryos toward the site of implantation. Any change in the characteristics of a uterine contraction directly affects intrauterine fluid flow and, consequently, the trajectories of embryo movement toward the site of implantation are altered.

Moreover, the uterus may be irritated by the insertion of the catheter into its cavity or its contact with the fundus and may respond by increasing the number or amplitude of contractions in real clinical ET operations [32, 33]. In this study, uterine contraction, as a possible source of embryo propulsion in the uterus, was not present and it is not known whether fluid flow in the simulated transfers mimics *in vivo* clinical circumstances or accurately reflects intrauterine fluid flow. In future research, the application of soft materials to construct a three-dimensional in vitro uterine model is suggested. The combination of a computer program with the model could simulate a contraction wave to better simulate the real physical conditions of the uterus. Thus, the movement tracking of embryos under uterine contraction would offer more meaningful information for the improvement of embryo transfer techniques.

## Conclusions

The *in vitro* laboratory mock system could provide a better understanding of the scientific phenomena that occur within the uterus due to controllable parameters of the ET protocol. Contrary to several clinical reports that embryo location may be found using the position of the air bubble under ultrasound-guided ET, there is a discrepancy between the location of the air bubble and the embryo in about 30% of cases. A fast-speed injection in a retroverted uterus is associated with a greater possibility of non-conformity between the location of air bubbles and embryos.

Finally, the ET model provides a new method to investigate ET procedures, such as different types of transfer catheters or embryo loading, which cannot be performed *in vivo* due to ethical and practical limitations.

## Supporting information

S1 Data (XLSX)

S2 Data (XLSX)
